# Clay Ingestion During Pregnancy Among Black African Women in a North London Borough: Understanding Cultural Meanings, Integrating Indigenous and Biomedical Knowledge Systems

**DOI:** 10.3389/fsoc.2020.00020

**Published:** 2020-04-07

**Authors:** Cathrine Madziva, Martha Judith Chinouya

**Affiliations:** ^1^Department of Health, London Metropolitan University, London, United Kingdom; ^2^School of Tropical Medicine, Liverpool School of Tropical Medicine, Liverpool, United Kingdom

**Keywords:** clay ingestion during pregnancy, Black African Women, cultural practice, indigenous knowledge, biomedical science

## Abstract

Findings from this qualitative audit conducted in a North London Borough among Black African women show that clay ingestion during pregnancy is a cultural phenomenon embedded in indigenous knowledge (IK). Reasons for clay ingestion include curbing morning sickness, nausea, satisfying cravings, “mineral deficiency” and other life sustaining beliefs. However, Public Health practitioners' top down approach and response which considers the practice as “dangerous” and potentially harmful to the health of the woman and unborn child with midwives and General Practitioner doctors called upon to discourage it, risks alienating the target population. Furthermore, within such a top down framework, opportunities to integrate biomedical science and indigenous knowledge systems are potentially missed. The use of culturally sensitive Public health interventions which consider a community approach, while attempting to integrate these two knowledge systems through further research is likely to bear more fruits.

## Introduction and Background

In 2013, Public Health England (PHE) published a press statement and directive to General Practitioner doctors (GPs), Directors of Public Health and other relevant staff to dissuade pregnant women from Asian and African communities from ingesting a potentially poisonous product known as calabash chalk (Public Health England., 2013). This was a follow-up alert after the UK Food Standards Authority (FSA) issued repeated warnings in 2012 and 2011 following the dictation of high levels of lead and arsenic in calabash chalk intended for ingestion and “detoxing therapies” sold by a number of online retailers (Food Standards Agency., 2012). While there is evidence of ingestion among Asian migrants in the 1980's in the UK (Middleton, 1989), an active campaign against calabash chalk ingestion appears to have started in 2002 with the FSA issuing its first official warning (Food Standards Agency., 2002, 2006). While it is not clear how much calabash chalk is entering the UK, in 2006, three consignments were prevented from entering the country, with posters and leaflets distributed to GP surgeries and primary health care trusts to discourage ingestion (Food Standards Agency., 2006). Environmental health officers have been at the forefront of seizing and removing this product from sale in London Boroughs such as Hackney (Bartholomew, 2013) and researchers such as Abrahams et al. (2006) and Al-Rmalli et al. (2010) have played a key role in informing FSA alerts by testing samples openly sold in “ethnic” shops in Birmingham, Leicester, Luton and East London. On its website, the agency currently advises pregnant and breast feeding women not to ingest clay, while adding that it is “sometimes” consumed by African and Asian communities because it may contain highly toxic chemicals (Food Standards Agency., 2018).

This is not without good reason; extensive scientific evidence suggests that persistent exposure to high levels of lead and arsenic found in the product, during pregnancy can lead to negative health outcomes for the baby such as low birth weight, impaired intrauterine growth, impaired neurodevelopment and intestinal blockages (Al-Rmalli et al., 2010; Reeuwijk et al., 2013; Nyanza et al., 2014; Frazzoli et al., 2016; Gundacker et al., 2017). Among African and Asian communities in the UK, calabash chalk is also known as Argile, Mabele, Nzu, Shiley, La Craie, Calabash clay, Pembe, and Kichungu (Public Health England., 2013; Nyanza et al., 2014). In this paper, we refer to this product as “clay.” Despite these concerns, clay ingestion, also known as geophagy (Njiru et al., 2011) remains an important aspect of pregnancy among African communities, who are the central focus of this paper.

In African contexts, ingested clay is taken from anthills, termite mounds on tree trunks, walls of traditional houses and dug out from rural mines and dry river basins (Reeuwijk et al., 2013; Hunter–Adams, 2016; Gundacker et al., 2017). In countries, such as Ghana and Nigeria, the mining of clay for ingestion in large volumes for sale at markets is a source of livelihood sustenance for many communities (Frazzoli et al., 2016) with cycles of cleaning, baking, shaping and cooking (Henry and Cring, 2013). While most of the clay is naturally occurring before this process, there is evidence of artificial forms prepared by mixing clay with wood ash and salt, in some cases animal fat followed by molding and baking or left to dry naturally (Sing and Sing, 2010). Baked clay is often shaped into blocks, tablets, small balls or sticks and sold at markets in countries such as Ghana, Nigeria, Tanzania, Congo, Cameroon as well as ethnic minority shops in Europe (Abrahams et al., 2006; Reeuwijk et al., 2013; Nyanza et al., 2014). However, it is noteworthy that not all clay is considered good enough to eat, particularly surface clay which may contain undesirables such as animal and human feces and other biological elements. Instead, clay is carefully selected “on the basis of appearance, texture and taste and excavated from well-known traditional sources” (Frazzoli et al., 2016, p. 1466). Indeed, some studies have pointed to the nutritional benefits of clay ingestion (Tayie et al., 2013). However, as highlighted prior some clay ingested by pregnant women has been shown to contain metals which are toxic to humans depending on the quantities consumed.

Despite the dangers associated with clay ingestion, there is evidence of this practice in humans dating back to centuries in different cultures, including Europe in the Seventeenth–Eighteenth centuries (Von Humboldt, 1872). Greek and Roman historical medical texts provide ample evidence of this, with Hippocrates (460–380 BC) making a reference to geophagy in pregnancy (Young, 2011) while Celsus (14–37 AD) links geophagy to anemia (Woywodt and Kiss, 2002). Evidence retrieved at prehistoric geographical locations between Tanzania and Zambia (Clark, 2001) is often cited to support the assertion that geophagy originated in Africa (Laufer, 1930) thereafter spreading to other parts of the world through slavery, (Izugbara, 2003) and globalization (Njiru et al., 2011). Geophagy is also widely practiced amongst pregnant women in other African countries including Zimbabwe, South Africa, Ivory Coast, Kenya, Zambia inter alia (Reeuwijk et al., 2013; Nyanza et al., 2014; Hunter–Adams, 2016).

Against a backdrop of migration, evidence of geophagy among migrant pregnant African women living in high income countries such as UK, Austria, the Netherlands and Belgium (Al-Rmalli et al., 2010; Reeuwijk et al., 2013) is not surprising. The discovering of this practice in this sub population group (Asian women included) in these countries, e.g., UK and the Netherlands evoked the interests of biomedical science to investigate the contents of the ingested clay, hence the extensive scientific evidence alluding to its dangers. Noteworthy is that the FSA's repeated warnings also echo that of the Dutch Food Agency in the Netherlands which, after sampling 13 clay products from Africa and Suriname sold on Dutch markets between 2004 and 2012, dissuades ingestion among pregnant women (Reeuwijk et al., 2013). Despite the repeated health warnings by PHE and the FSA, there is evidence that women continue to purchase and ingest clay (Diebelius, 2018). This suggests the current public health approach is having limited impact.

Policy makers and researchers have predominantly focused on clay ingestion as a dangerous practice which must be discouraged. At a national level, in England, this has resulted in the scarcity of research which explores the social and cultural dimensions of this practice among migrant black African women. Hence, a limited understanding of the underpinning rationale behind clay ingestion, has consequently led to a failed public health intervention. The intersectionality between ethnicity, pregnancy and clay ingestion in a context, such as England, where the latter is viewed as dangerous makes discussions about this a taboo subject. This suggests that expectant women will ingest clay in silence, thereby threatening the potential of discursive dialogue between biomedicine and IK. In this paper, we reflect on clay ingestion in pregnancy as a cultural practice, an aspect of IK systems among black African women and how this conflicts with the biomedical science consequently leading to a failed Public health intervention in England. We begin by engaging with historical perspectives which have shaped geophagy, followed by a theoretical framework.

## Historical Perspectives on Geophagy: The Medical Gaze and Colonial Gaze

While clay ingestion in pregnancy is culturally embedded and widely acceptable with a prevalence of 84% observed in some African countries, in western societies, particularly in biomedical circles, it is viewed as a “shameful and highly suspect behavior limited to the deprived” (Njiru et al., 2011, p. 2). This viewpoint is rooted in two perspectives which have shaped the geophagy discourse, i.e., the colonial gaze and medical gaze. Coined by Foucault, the medical gaze refers to the medicalization process “by which non-medical problems become defined and treated as medical problems usually in terms of illness and disorders” (Tischler, 1999, p. 550). In a quest to understand and deal with behaviors and conditions deemed socially undesirable, such as geophagy, Henry and Cring (2013) posit that scientists and physicians, medicalise these, with geophagists labeled patients. Based on observations of African slaves on Caribbean and American plantations by plantation owners and European physicians, literature from the Eighteenth and Nineteenth centuries portray geophagy as a disgraceful practice among the “savage” linked to disease and ill health (Higman, 1984; Kiple, 1984; Lacey, 1990; Horner et al., 1991). Among perceived clay “addicts,” identified symptoms ranged from “sluggish… depression, shortness of breath, abdominal swelling and melancholy” which ultimately led to death (Henry and Cring, 2013, p. 187). Hence, out of great concern for slave derived profits, some plantation owners had facial masks fitted on their slaves to stop clay ingestion (Woywodt and Kiss, 2002).

Rooted in the colonial enterprise, Pratt (1992) defines the colonial gaze as the self-given right by European colonizers to decree, name and control reality in countries they colonized. Through the colonial gaze, colonizing Europeans also shaped the geophagy discourse, through moralization and the social construction of the “other” (Henry and Cring, 2013). Driven by an air of superiority, colonial explorers and administrators depicted geophagy as a deprived appetite associated with uncivilized “other,” i.e., African and South American natives. Within the post-colonial era, biomedical preconceptions continue to shape the geophagy discourse, with the medical gaze emphasizing the disease aspect. As already highlighted, ingested clay is analyzed for the presence of metals such as lead and arsenic, with potential health implications spelt out (Kutalek et al., 2010; Gundacker et al., 2017). On one hand, holistic anthropological perspectives consider clay ingestion as a mineral supplementation in some instances (Frazzoli et al., 2016) and a remedy for appetite challenges along with nausea, morning sickness and salivation during pregnancy. However, it is important to recognize that in tandem with economic development, the disposal of hazardous materials and chemicals, and mining activities have contaminated clay which potentially ends up ingested thereby posing health risks. Hence, it remains vital to analyze clay for toxicity. Nevertheless, the stigmatization of its' ingestion as a dangerous and shameful bahaviour poses challenges for Public Health practitioners' engagement with Black African pregnant women in antenatal care practice. Against this background, this paper aims to bring about a more nuanced understanding of clay ingestion during pregnancy within a cultural framework as a starting point to finding ways of integrating IK and biomedicine knowledge, while offering recommendations on the way forward.

## Theoretical Framework: A Cultural Practice Embedded in Indigenous Knowledge

Known as traditional knowledge and local knowledge, Warren et al. (1991) posits that IK is unique to a given society and forms the basis for decision making at local levels in relation to activities such as health care inter alia. In this paper, we define IK as long standing traditions, practices and beliefs “generated, refined, and passed” from generation to generation in a given society which inform decision making regarding life sustaining activities thereby forming an integral part of the society's cultural identity (Bag and Pramanik, 2012, p. 8). On this note, a key distinction often made about IK, in comparison to biomedical knowledge generated through scientific research, is that “it does not separate secular and rational knowledge from spiritual knowledge, intuitions and wisdom … [and] distinction between intangible knowledge and physical things is often blurred. It [IK] cannot be divorced from the … cultural context within which it is arisen …” (Kothari, 2007, p. 4). This propels the argument that IK is deeply embedded in a society's cultural values and practices. As postulated by Chinouya and Madziva (2017), culture is often a crucial determinant of reproductive health behaviors including those relating to accessing antenatal care within the timeframes prescribed by clinicians, namely 12 weeks into the pregnancy.

Helman (1984, p. 3) defines culture as “a set of guidelines (explicit and implicit) which an individual inherits as a member of society, and which tells him how to view the world and how to behave in it in relation to other people, to supernatural forces or gods and the environment…the inherited “lens” through which the individual perceives and understand the world.” Similarly, Swidler (1986) argues that culture provides a toolkit for a society's world view, from which they select lines of action, which shape their behaviors. Against this backdrop, the heterogeneity of African IK systems notwithstanding, there is evidence that clay ingestion during pregnancy is an imbedded cultural practice shared by many African societies (Njiru et al., 2011; Diko and Diko, 2013; Henry and Cring, 2013; Reeuwijk et al., 2013; Frazzoli et al., 2016). Culture therefore plays a crucial role in informing Black African women's world views as they seek the best outcomes for their unborn babies and selves. Situating clay ingestion around this shared cultural framework therefore enables us to make sense of the practice as well as making appropriate recommendations to Public health practitioners in antenatal care.

In the context of migration, studies show that migrant women hold on to practices and beliefs about pregnancy passed on to them by past generations from countries of origin (Benza and Liamputtong, 2014; Cousik and Hickey, 2016; Quintanilha et al., 2016). Black African migrant women in this London Borough, similarly, brought to the host nation clay ingestion, a cultural practice previously utilized in the management of pregnancy in everyday lives in their “homeland.” However, this poses challenges for antenatal care Public Health practitioners who consider the practice undesirable and dangerous. Thus, as part of the “other” culture which shapes some black African women's reproductive bahaviours, clay ingestion is dismissed as a public health menace without due regard to its social and cultural dimensions. We argue that this approach which is rooted in the medical and colonial gaze results in ineffective public health interventions, as well as missed opportunities to integrate IK and biomedicine. Moreover, clinical guidance about clay ingestion is grounded in scientific “facts” which informs the medical gaze. However, as (Lupton, 1997) (cited in Chinouya and Madziva, 2017, p. 30) argues “cultural understandings of the body, health and the causes of disease are all integral to the epidemiological construction of ‘facts' for clinical guidance.” Based on findings from a health audit commissioned by Public Health Practitioners in a North London borough, in this paper, we reflect on clay ingestion during pregnancy informed by the “other” culture of Black African women. We do so as a starting point to finding ways of integrating knowledge from IK and biomedicine for the delivery of culturally sensitive Public health interventions in antenatal care in England.

## Study Context

This was part of a larger audit study, using qualitative approaches, which aimed to improve antenatal care by public health specialists in this North London borough by seeking to understand some barriers and facilitators to the booking appointment within the prescribed 13 weeks of gestation among Black African women who are over represented in the proportion of “late bookers.” Ingesting clay emerged as an important aspect of pregnancy and was explored in detail during the interviews. Study participants repeatedly raised and discussed geophagy in relation to understanding the meanings of pregnancy and the precautions taken in the context cultural beliefs embedded in IK. Researchers visited African markets and shops and observed a wide range of foods imported from African countries—including clay, which was often on display or in some cases, out of sight with customers having to ask if clay was available. Indeed, discussions with some sellers revealed that in some cases, they probably saw pregnant women, first before the women went to visit the midwives for the booking appointment. Most African women in this setting were identified as “late bookers” who made first clinical, antenatal presentations well after the recommended, 12 weeks of pregnancy.

## Methods

A qualitative approach was chosen as this allows deeper exploration of the broader cultural factors associated with pregnancy including “eating clay” among black African women in this North London borough. Semi-structured Interviews were used thus enabling deeper insights into the women's experiences (Ritchie and Lewis, 2003). Participants were recruited using snowballing and opportunistic sampling in a number of community-based settings including African community organizations, churches, shops, and community centers. Despite the potential risk of recruiting a particular “type” (Griffiths et al., 1993), snowballing enabled us to recruit hard to reach participants who might otherwise not have taken part in the audit. Given the “hidden” nature of the target population some potential participants were reluctant to take part due to fear that the project was connected to the UK Immigration Department.

## Participants and Ethical Considerations

The participants who took part in the original study perceived themselves as exerting an influence on how black African women in their networks and families make sense of pregnancies as well as accessing health services. In addition, participants had to self-identify as above age 18, Black African, resident in this North London borough with experience of using maternity services in the borough.

Twenty- three participants who self-identified as Black African took part in the original audit and more than half of these made references to clay ingestion in the context of cultural practices associated with pregnancy. Participants identified themselves as mothers and first-generation migrants from Ghana, Kenya, Uganda, Zimbabwe, and Nigeria. Guided by the NHS Health Research Authority and the UK Policy Framework for Health and Social Care on Services Evaluation, the commissioning North London Public Health Authority defined the parameters of the study as an audit. Ethical approval was therefore not required as the Health Research Authority Decision Tool judged the study to be an audit, not research. After briefings were held between us the researchers, and the commissioning Public Health strategist we designed an interview schedule in line with data the audit aimed to elicit. In tune with research protocols, we briefed all participants regarding the aims of the audit, issues of consent, confidentiality, voluntary participation and right to withdraw from the study. Informed consent was obtained from all participants who voluntarily agreed to take part. To ensure confidentiality all participants were numbered while retaining country of origin.

We recorded all interviews and transcribed these followed by an analysis using Thematic Framework in which units of meaning were grouped into themes (Ritchie and Lewis, 2003). Themes around “eating clay” during pregnancy emerged as follows: (a) cultural practice (b) managing nausea (c) craving (d) life giving.

## Results

### Clay Ingestion in Pregnancy: A Cultural Practice

Participants reported that during pregnancy, there were certain things women particularly from the African continent were supposed to eat. Clay was the first thing mentioned as noted below:

In terms of our culture, there are certain things that you are expected to eat and not to eat. You are expected to eat soil [clay]. This is because when you are pregnant and it rains the smell of rain and hormones make you feel like eating soil… (Kenyan mother 3)

Clay eating during pregnancy was normal and known in her culture and she put it this way:

Everyone knows pregnant women eat clay in my culture. When they see, a woman eating clay, they will say, ahhh, you are now pregnant! Even though some people who are not pregnant eat it. When I was pregnant, I would send my husband to get me the clay. (Ugandan mother 1)

As expressed above, clay ingestion in pregnancy was a phenomenon which participants situated in culture, and as such was readily accepted as well as expected by most people. Her husband, a key figure in decision making with regards to the pregnancy, was also an enabler in the practice. Though not just only confined to pregnant women, clay ingestion was often interpreted culturally, as a sign of pregnancy before this was potentially confirmed by doctors, midwives or off the counter-pregnancy testing kits. In addition, clay ingestion was viewed as cultural practice passed from one generation to another as one participant put it this way:

My aunts, mother and pregnant neighbors ate clay back home (Kenya). You see for generations after generation, pregnant women have been eating clay. (Kenyan mother 10)

Another participant noted that eating “soil” during pregnancy was ubiquitous within the African continent, the heterogeneous nature of African cultures withstanding:

It happens (clay ingestion) in Uganda or all over Africa… when you don't eat it you look pale. We buy it here… the clay, we buy it, they sell it openly. (Ugandan Mother 4)

As noted by the Ugandan mother above, not eating clay in pregnancy had consequences that included “looking ‘pale' which can be interpreted as ‘looking unwell'.” It was also noted that clay was easily available in this North London borough and one could easily buy this from the “shops.” Researchers visited one of the “shops” and the seller declined to be interviewed but mentioned in passing that he does see a lot of pregnant African women. However, in some instances clay was brought over from Africa by family members who often traveled there as noted below:

My daughter, she goes back home (Kenya) often so she brings it for me. Last time, she brought this clay; I didn't like it that much. It didn't have the normal taste that I like. So, I ate it a bit and threw it away because it didn't taste right. (Kenyan mother 10)

Throwing away of the clay which “didn't taste right” suggests that not just any clay is ingested, i.e., it must have a certain acceptable taste.

## Craving

Clay was however just not sold in this North London borough, but “back home” as well to satisfy the huge demand brought on by pregnant women who craved for it as noted below:

Back home in Ghana, clay is sold at most markets. You can get different types there; it's big, big business because most pregnant women crave for it. Even those not pregnant, some just have a craving. They bake it and package it nicely in plastics like sweets. (Ghanaian mother 11)

As the quote above suggests, desire to satisfy cravings was one of the key reasons associated with clay ingestion. The idea that clay was now a “big business” alludes to its commercialization in contrast with the excerpt below where the participant makes reference to individuals taking the clay from its original source, albeit equally driven by cravings:

When pregnant, you crave for certain things and I would say some of us eat clay because we crave for it. White women here crave for other things, but for us, it's clay. Because we crave for clay, it should not be seen as something to be shamed about. The main concern should be about the source of clay, because some people now buy and sell it. At home, you can go take it from a well-known source … we have ant hills, they have the best clay. And it is safe, our ancestors ate clay in their pregnancies without anything bad happening to them. (Zimbabwean mother 6)

According to the Zimbabwean woman above, “nothing bad” happened to their ancestors who also ate clay during pregnancy. Others made links between cravings and nutrient deficiencies as noted:

When you eat the clay after a craving, you will be satisfied but when you look at the books, it says that you feel like that because you have a deficiency in some nutrients, so the clay helps you there. (Kenyan mother 7)

There was a recognition that while “white” women craved for other things, as Africans, they craved for clay. However, alluding to clay ingestion as something not to be ashamed of indicates an awareness that this was viewed as a shameful practice in this context, unlike “back home” where everyone knew pregnant women ate clay as indicated in previous quotes. Furthermore, the participant went on to defend the practice as safe (without being prompted about safety issues) against a backdrop of this being a behavior practiced by their ancestors. As also noted above, some believed that the craving was brought on by a deficiency in nutrients which was complemented by what they believed was in the clay. This resonates with idea of “looking pale” when one does not ingest clay as previously indicated by one of the participants. Another pointed out that the midwives “back home” were also partakers, hence didn't dissuade pregnant women from the practice:

Even the midwives don't tell you not to eat clay (in Nigeria), because they are also women, and when pregnant, they also eat it like everyone else. They also get the craving. (Nigeria mother 9)

While the midwives referred to above could be traditional ones as opposed hospital midwives, this nevertheless shows how widely acceptable the practice is. For another participant, the craving was so strong to the extent of waking up just to ingest as noted below:

It started as a habit, but also a craving, but as a pregnant woman, I ate more clay than before. Such that if the craving came upon me in the middle of the night in bed, I would wake up for a bite (laughs). It was always next to my bed side; the craving was really strong. (Ghanaian Mother 12)

While the majority of women acknowledged eating clay or knowing someone who did as a remedy for cravings, a participant noted that it was often sourced from trusted communal places and which resonates with another participant who referred to anthills as follows:

When we were growing up (in Zimbabwe), there was an ant hill nearby. An elderly woman lived close to it. Everyone called her grandmother as we do, most pregnant women used to go to that anthill to get clay. That anthill was well-known for its clay because it was tasty. Back then my mother would send me to get her some clay there, but before getting home, I would eat some because it was so good. No one would go there to do their toilet business; it was a special place. (Zimbabwean Mother 6)

Another participant worried about the commercialization of clay because market sellers were not potentially concerned about their customers but making money:

There is need to take care now because things have changed. It is now more crucial for the clay to come from the right place. When things were not commercialized, and clay was free, you knew it was safe. But now with it has become commercialized, you just don't know. People are eager to make money; they don't care about what happens afterwards to pregnant women. (Ugandan mother 2)

While clay was widely ingested, only one participant among the dozen who made references to clay in the study expressed concern about its safety without being prompted which raises questions about women's knowledge and awareness of the potential risks associated with the practice.

## Nausea Management

In addition to satisfying cravings, clay was ingested to manage nausea among other things. Two participants put it this way:

There are many things pregnant women eat to rid of morning sickness, some ate lemons, some eat the skin of oranges but clay is an all-rounder. It can beat morning sickness; it can also be for the craving and for the minerals and vitamins that someone might lack. (Nigerian Mother 13)

Another one said:

You see pregnancy can be a difficult time, especially the first 3 months. Some people have no energy to do anything. Some people experience so much nausea and clay helps with that. But you should not eat too much because of constipation. You must drink plenty of water too. (Ugandan mother 4)

As the first quote suggests, clay was perceived as “all-rounder” because it helped to manage morning sickness, the cravings and provided vitamins, better than lemons and orange skin. However, if eaten in large quantities there was an awareness of risking constipation.

## Life Giving

In addition to all above, clay was considered life giving, with religious connotations regarding the beginning of life perceived through creation, and returning to the soil in death. This participant put it this way:

Everything comes from soil; life comes from the soil. Are we not created from soil? And when we die, we go back to the soil, so there is nothing strange about eating it. Look at the way plants planted in rich clay soils do well and the ones planted in sandy soils don't do well. We too eat the good rich clay, not just any soil. It gives life. (Kenyan Mother 14)

Because “everything comes from soil” ingesting clay was considered normal. As much as plants require good rich clays to sustain them, so did the women, particularly during pregnancy when sufficient nutrients are required to sustain the fetus. However, only the “right” kind of clay was perceived as giving life. Another participant perceived clay as lifesaving during sickness brought on by a traumatic pregnancy:

See, there was a time I was so sick back home (Ghana). I was so sick … a difficult pregnancy. I went back to work just because I had to, and the only thing which kept me alive was clay. I did not have an appetite, nothing. I would eat the clay and drink some water and go to bed. It was the only thing I could eat till I was strong, till my appetite came back…it saved my life. (Ghanaian mother 12)

When one participant was asked if she had disclosed clay ingestion at the booking appointment, she responded:

No, you can't tell them (GP/ midwives) you are this person who is eating clay or washing in some leaves from back home (Uganda) to keep the baby healthy (laughs). They will think you are crazy, so you don't talk about those things. (Ugandan Mother)

The reluctance to talk about clay ingestion could be potentially related to the idea of a “shameful practice,” with some concern about the practice attracting stigma among medical professionals.

## Discussion

Findings from this study echo results from elsewhere which suggest that clay ingestion during pregnancy among black African women is widespread among African societies (Kutalek et al., 2010; Frazzoli et al., 2016; Gundacker et al., 2017). In this study and literature (Njiru et al., 2011; Nyanza et al., 2014), the association between pregnancy and clay ingestion was perceived as normal, with the latter often viewed as the first sign of pregnancy. Upon migration, women who participated in the study brought clay ingestion knowledge to England, in line with findings from other studies which show the practice has been introduced by migrants to UK and other western nations (Abrahams et al., 2006; Al-Rmalli et al., 2010; Reeuwijk et al., 2013), thereby highlighting its social dimension (Henry and Cring, 2013). Beyond this social dimension, participants in this study situated clay ingestion during pregnancy as a cultural practice passed down from past generations which concurs with findings by Frazzoli et al. (2016) which identify this as a cultural heritage among African societies in Zambia, Cameron, Tanzania, Nigeria, South Africa, and Kenya inter alia. Indeed, a number of research studies have identified clay ingestion as deeply embedded in indigenous practices and or cultural practices or cultural variables (Geissler et al., 1999; Njiru et al., 2011; Diko and Diko, 2013; Henry and Cring, 2013; Reeuwijk et al., 2013; Frazzoli et al., 2016). This affirms the notion that culture provides a toolkit for a society's world view, with knowledge being passed down from generation to another which shape their behaviors (Swidler, 1986). Against this background, the role of culture in shaping Black African women's reproductive health behaviors with regards to clay ingestion cannot be ignored.

While the commodification of ingested clay “back home,” where previously not the case, worried some participants, this contrasted sharply with the acceptance of its widespread commodification in countries such as Ghana and Nigeria which resonates with study findings from these countries inter alia (Nyanza et al., 2014; Frazzoli et al., 2016). In the context of migration, as researchers we observed clay imported from Africa being sold in African shops in London as recorded elsewhere in Europe (Abrahams et al., 2006; Reeuwijk et al., 2013). The continuous ingestion of clay by Black African women in this North London Borough through either purchasing or relying on those visiting “back home” to bring it, suggests of “actors selecting continuity from their cultural toolkit” (Chinouya and Okeefe, 2006, p. 96) as passed down from past generations in countries of origin. A review of qualitative studies by Benza and Liamputtong (2014) which explored the experiences of pregnancy amongst migrants in 11 Western European countries, similarly found that a complex cultural framework of values and beliefs from countries of origin influence women's health behaviors in host countries. Against this background, Black African migrant women who view clay ingestion as an important aspect of pregnancy, may not listen to the top down warnings from Public Health England.

The medicalisation of pregnancy treats women as “vessels,” devoid of spiritual health that can benefit the mother and her baby. In this study, ingesting clay was described as an “all-rounder,” i.e., connecting life and death through women's bodies. In tune with findings by Diko and Diko (2013) where clay ingestion was associated with rituals and beliefs and cosmology and symbolism, in this study, clay was considered life giving, with religious connotations made between the biblical beginning of life through creation as well as death, when bodies return to the earth. This reinforces the notion of a cultural practice embedded in IK which often does not separate rational knowledge from intuitions, spiritual knowledge and wisdom with blurred boundaries between the tangible and intangible things. Taking a holistic approach to women's health during pregnancy and the knowledge they bring during clinical encounters with midwives and GPs should facilitate nuanced discussions about their health and well-being during pregnancy.

Faced with cravings, clay ingestion was perceived as a mineral supplement which catered for potential deficiencies, hence not ingesting clay was associated with “looking pale” which resonates with literature (Abrahams and Parsons, 1996). While pregnancy cravings were considered normal, with “white women” perceived to satisfy these through other means, there was awareness that clay ingestion among “us.” i.e., black African women was perceived negatively. Similar studies have explored the relationship between the body, health and pregnancy, including the early work of Obeyesekere (1963) in Sri Lanka which showed that pregnancy cravings in the given context were culturally and socially determined. Furthermore, the idea that ingesting clay was nothing to be ashamed of as raised by a participant indicated an awareness of the stigma associated with the practice unlike “back home” where in some communities, none ingestion during pregnancy was considered strange (Njiru et al., 2011). Thus, viewed through the lenses of the “other” culture which shapes black African women's health behaviors, ingestion was perceived a normal cultural expectation as well as a socially sanctioned habit which concurs with findings from other research studies (Njiru et al., 2011; Frazzoli et al., 2016; Hunter–Adams, 2016).

In the context of antenatal care practice, there was evidence that women were unlikely to disclose clay ingestion, along with the use of other traditional products such as “leaves from back home” to health care professionals because they worried being perceived “crazy” as noted by a Ugandan mother. This supports the assertion by Njiru et al. (2011) that the perception of clay ingestion as a shameful behavior leads to under reporting in health care settings by pregnant women. While in this instance, clay is perceived a food product, the hegemonic link between the notion of “dirt” which is a social construction, i.e., dirt being garbage out of place and soil (Douglas, 1966) compounds stigma issues. Hence, the lack of disclosure in antenatal care practice suggests that expectant women will continue to ingest clay in silence. Thus, positioning clay as a danger to women and their unborn babies and as a subject that is to be addressed with a top down approach can only strengthen the silence and its position as a taboo subject. This creates challenges for midwives and GPs in particular as targeting pregnant African women to discuss the risks posed by clay ingestion could be construed as racist.

How can knowledge from this study and elsewhere which indicate that clay is ingested as a remedy for morning sickness and nausea among other life sustaining beliefs (Njiru et al., 2011; Frazzoli et al., 2016) be integrated with the evidence from biomedical science which indicates that some clays pose danger? We contend there is need for nuanced understanding, particularly on the part of Public Health practitioners, that alerting women to the dangers of ingesting clay without understanding its cultural and social roots, potentially acts as a stumbling block to the delivery of effective and culturally sensitive public health interventions. Furthermore, this hinders the exploration of innovative pathways to integrating biomedical knowledge and IK regarding the practice. More than 40 years, ago; Bradford Council in England produced “an eye- catching leaflet stating that sikor (clay) may be consumed as mineral nutrient supplement” (Middleton (1989) cited in Abrahams et al., 2006, p. 99). Could such an approach work against a backdrop of scientific tests and checks on clay products to demonstrate safety for human consumption?

Despite the repeated warnings by the FSA and PHE to pregnant women to stop ingesting clay, evidence from this study and elsewhere (Diebelius, 2018) show that women continue to purchase and ingest clay. Furthermore, the commodification of ingested clay by online retailers continues to gain momentum. A search on the online retail giant Amazon, brings up a number of clay products with some sellers touting their safety for internal and external use as well as buyers signaling product satisfaction with mostly 5-star ratings. These stark ground realities suggest there is need to rethink the top down public health approach. In one of its notifications, the (Food Standards Agency., 2012) aptly warns that clay products are not regulated in the UK; hence the ingredients cannot be monitored or controlled. Given the rise of clay commodification and the continuation of this practice despite repeated warnings, is it not time to consider appropriate regulation, with safe clay products, if any, certified for fitness for human consumption?

From an anthropological perspective, there is recognition of health benefits associated with clay ingestion under certain circumstances (Abrahams and Parsons, 1996; Henry and Cring, 2013; Tayie et al., 2013). However, because the chemical composition of clay differs greatly depending on the source, this makes it difficult to assess either potential dangers or benefits to health posed by all clay. As already highlighted elsewhere, there are changes in living environments which pose risk factors to ingesting clay e.g., waste disposal, mining activities and biomedical science has been most effective in pointing these out. Given these changes, a call for this cultural practice to be revisited is in order. In the words of Frazzoli et al. (2016, p. 1465) “therefore benefits to risks ratio of cultural behaviors initiated centuries ago based on traditional medical practices require deep revision and assessment.” This raises the question: How can this be done without continuing to marginalize the “other” cultural practices and beliefs, “often figured as inferior forms of knowing to be replaced by universalized knowledge derived from the Western scientific tradition” (Bag and Pramanik, 2012, p. 8)?

As a starting point, we note that most research studies focus on clay toxicity and potential health risks, hence the practice is viewed as shameful and savage; a psychological disorder which must be banished. While understanding clay toxicity is pivotal, the potential health benefits associated with clay ingestion in specific circumstances remain under researched. This skewed knowledge base has resulted in neglect by policy makers, hence the lack of appropriate regulation and blanket Public Health campaigns to dissuade pregnant women from the practice. We argue that there is need for future research studies to consider health claims associated with none toxic clay with the aim of identifying clay fit for human consumption, if any. This can potentially inform appropriate policy directions which is particularly important because despite the repeated health warnings, clay ingestion is a cultural practice that has survived for centuries, hence the likelihood of persisting for generations to come.

Lastly, clay ingestion amongst black African women should not be divorced from the other structural issues that exacerbate health inequalities, particularly given that the UK Public Health Outcome Framework takes the life course approach to maternal health improvement. An approach that Public Health England may wish to consider is to work very closely with community women's groups, networks, churches and other agencies. For example, African women are over represented in HIV prevalence and it has been shown in interventions that African communities can be engaged in public health initiatives if they feel that they “own” the problem and are given the platform to design interventions that sit well within their diverse cultures (Chinouya, 2004). In the absence of regulation and clay clearly certified as safe for human consumption, community-led initiatives which raise awareness about the potential dangers of clay ingestion in pregnancy are required because the current top down approach risks further pushing the practice underground. We call upon Public health authorities to work closely with community groups so they design bottom up, culturally competent interventions.

## Conclusion

In this paper, we considered clay ingestion within a cultural framework. Findings show that clay ingestion during pregnancy is a culturally imbedded common practice among black African women and in countries of origin. Women were however unlikely to disclose engaging in this practice due to the stigma associated with eating “dirt” in a context where this is deemed dangerous. Addressing potentially unsafe practices during pregnancy requires a community engagement approach as top down approaches risk alienating the target population. Hence we suggest using community groups as platforms for discussing potential risks associated with clay ingestion. However, as a starting point to integrating biomedical science knowledge and IK systems, research which considers, the claims associated with the health benefits of none toxic clay is required.

## Data Availability Statement

The datasets generated for this study are available on request to the corresponding author.

## Ethics Statement

Ethical review and approval was not required for the study on human participants in accordance with the local legislation and institutional requirements. The patients/participants provided their written informed consent to participate in this study.

## Author Contributions

CM and MC worked together in designing the work. They collaborated in developing the interview schedule, data collection, analysis and interpretation and authoring the paper. CM took a lead in the writing process.

### Conflict of Interest

The authors declare that the research was conducted in the absence of any commercial or financial relationships that could be construed as a potential conflict of interest.
